# Multiple thrombosis associated with Cytomegalovirus enterocolitis in an immunocompetent patient: a case report

**DOI:** 10.1186/s12879-021-06230-4

**Published:** 2021-06-05

**Authors:** Kaisei Kamatani, Tsuneaki Kenzaka, Ryu Sugimoto, Ayako Kumabe, Akihito Kitao, Hozuka Akita

**Affiliations:** 1 Department of Internal Medicine, Hyogo Prefectural Tamba Medical Center, 2002-7 Iso, Hikami-cho, Tamba, 669-3495 Japan; 2 Division of Community Medicine and Career Development, Kobe University Graduate School of Medicine, 2-1-5, Arata-cho, Hyogo-ku, Kobe, Hyogo 652-0032 Japan; 3 Department of General Medicine, Toyooka Public Hospital, 1094, Tobera, Toyooka, Hyogo 668-8501 Japan; 4 Division of Medical Oncology/Hematology, Department of Medicine, Kobe University Graduate School of Medicine, Kobe, 650-0017 Japan

**Keywords:** Cytomegalovirus, Thrombus, ADAMTS13, Thrombotic microangiopathy, Immunocompetent

## Abstract

**Background:**

Cytomegalovirus (CMV) is reported to have thrombogenic characteristics that activate factor X in vitro and stimulate the production of factor VIII and von Willebrand factor (vWF). Thrombosis associated with CMV infection is prevalent among immunocompromised patients and predominantly presents as a solitary large thrombus in the deep vein, pulmonary artery, splanchnic arteriovenous ducts, or other similar sites. Multiple thrombi, however, are rarely observed in such cases. Here, we report about an immunocompetent man with multiple microthrombi associated with CMV infection.

**Case presentation:**

A 72-year-old Japanese man who complained of abdominal pain was hospitalized with multiple colonic stenosis. He was later diagnosed with CMV enterocolitis and treated with ganciclover from Day 27 post-admission. During hospitalization, the patient developed thrombi in his fingers. He was initially treated with anticoagulant therapy (rivaroxaban); however, the therapy was discontinued owing to a prolonged activated thromboplastin time and an elevated international normalized ratio of prothrombin time. Instead, vitamin K and fresh-frozen plasma were administered. Nevertheless, his coagulation profile remained abnormal. Eventually, he developed colonic perforation and had to undergo emergency surgery. An intraoperative specimen showed several microthrombi in the middle and small arteriovenous ducts of his small and large intestines. The patient’s coagulopathy improved preoperatively, and his overall condition improved postoperatively. Since the activation of ADAMTS13 was reduced remarkably, the thrombotic tendency was determined to be a thrombotic microangiopathy-like condition owing to increased vWF. We could not attribute the coagulopathy to any other cause except CMV infection; therefore, we concluded that this was a case of multiple thrombosis associated with CMV.

**Conclusions:**

We present an extremely rare case of a patient with multiple thrombotic microangiopathy-like microthrombosis caused by CMV infection. Our findings suggest that CMV infection may be considered as a differential diagnosis for immunocompetent individuals who present with thrombosis of unspecified cause.

## Background

Infections caused by cytomegalovirus (CMV) are often asymptomatic; however, symptomatic manifestations of the infection vary from those of a mononucleosis-like syndrome to severe viral dissemination in immunocompromised patients [1]. Arteriovenous thrombosis is a rare complication of CMV infection and often presents as a solitary large thrombus in the deep veins, pulmonary arteries, or visceral arteriovenous ducts [1]. Multiple microthrombi consequent to CMV infection are rare, and only a few cases have been previously reported [2]. In addition, thrombosis associated with CMV infection often occurs in immunocompromised individuals [1] and has only recently been reported in immunocompetent patients [3].

We report the case of an immunocompetent patient who developed multiple microthrombosis subsequent to CMV colitis complicated with severe coagulation abnormalities.

## Case presentation

The patient is a 72-year-old man capable of independently performing activities of daily living. Regarding his medical history, he developed gastric ulceration at 18 years of age and underwent partial gastric resection. He was treated with rivaroxaban 10 mg / day for atrial fibrillation. Also, he regularly inhales formoterol fumarate for managing bronchial asthma. He used to smoke 20 cigarettes/day and drink more than three cups of sake between 20 and 65 years of age. He reported no positive history of food or drug allergy.

**Fig. 1:**
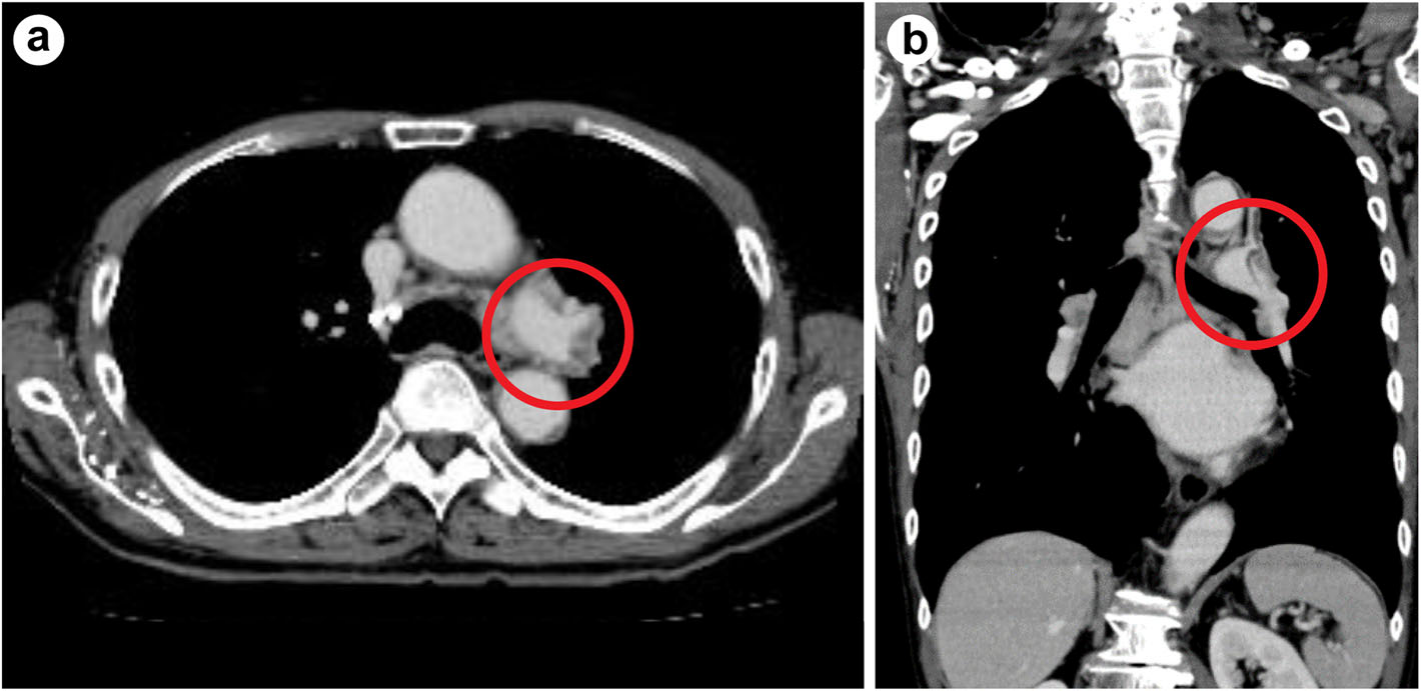
Thoracoabdominal contrast-enhanced CT on Day 14. The CT showed a contrast defect area in the main trunk of the left pulmonary artery

Approximately 40 days before admission to our department, the patient developed pain in the lower left abdominal region. Eight days later, he visited a hospital where he underwent non-contrast computed tomography (CT) of the abdomen that showed multiple colonic stenosis. Colon cancer was suspected, and the patient was admitted to the facility immediately. The patient also had fever (38 °C) on admission and, therefore, underwent thoracoabdominal contrast-enhanced CT that showed pulmonary thromboembolism (Fig. 1). Therefore, the dose of rivaroxaban was increased from 10 mg/day to 30 mg/day on Day 14 (days numbered from day of admission at the first facility).

**Fig. 2:**
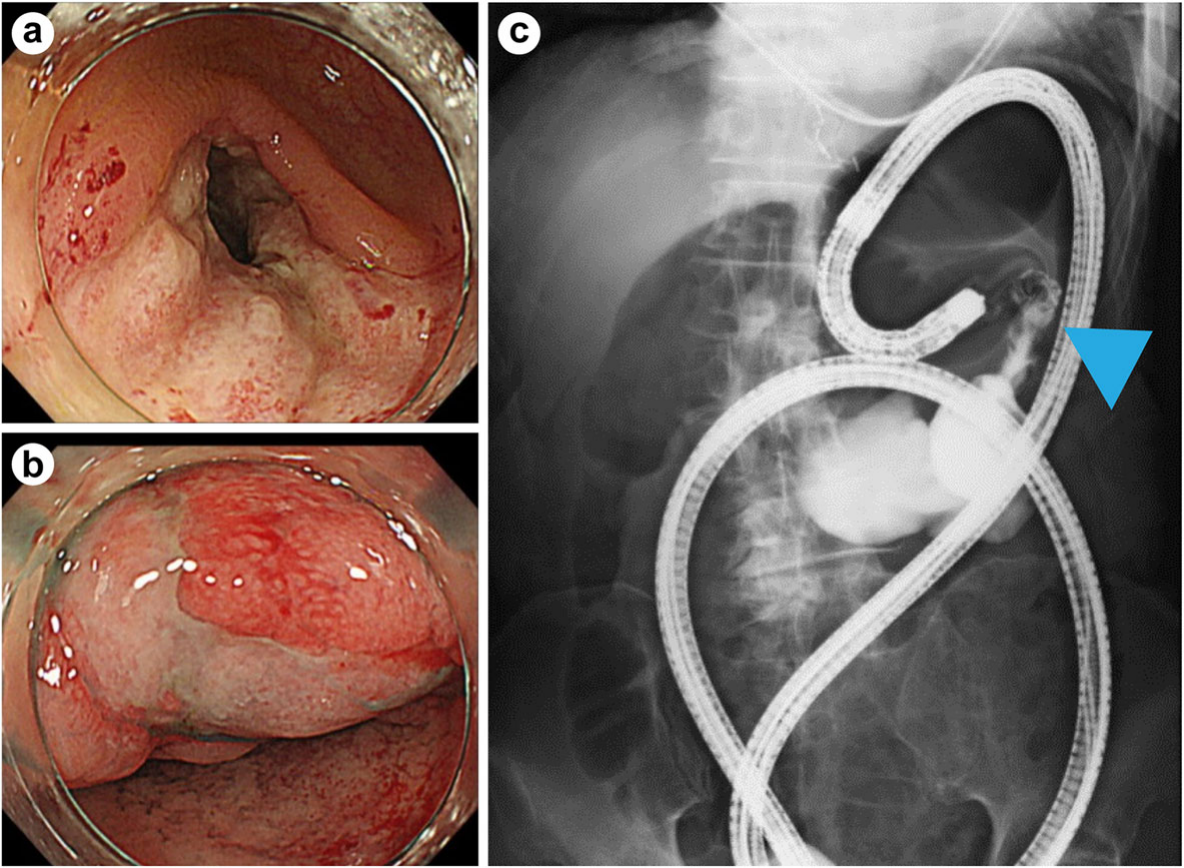
Colonoscopic intestinal findings on Day 14. **a** Stenosis in the hepatic flexure of the transverse colon, **b** ulcer in the sigmoid colon, and **c** 55-mm stenosis of the transverse descending colon (arrow) on contrast study

Then, he underwent fasting treatment and received maintenance intravenous fluids. On Day 14 of hospitalization, he underwent colonoscopy that showed stenosis in the hepatic flexure of the transverse colon and a circumferential ulcer of the sigmoid colon. On administration of a contrast, a 55-mm stenosis was found in the transverse and descending colon (Fig. 2). Pathological examination of the stenosed colon tissue revealed intranuclear inclusion bodies and a positive immunostaining for anti-CMV antibodies and CMV antigens (CMV DNA viral load was not performed). Consequently, the patient was administered 5 mg/kg ganciclovir (GCV) every 12 h since Day 27. Although the patient’s abdominal pain was relieved by the treatment, the fever was not alleviated.

Although the coagulation profile of the patient was normal on the day of admission (Day 1), an abnormal coagulation profile was seen on Day 25; the international normalized ratio of prothrombin time (PT-INR) was elevated (8.35) and the activated partial thromboplastin time (APTT) was prolonged (170.8 s). As a result, rivaroxaban was discontinued. Up till then, the patient had received a total of 18 units of fresh-frozen plasma (FFP) and 180 mg of vitamin K. However, the coagulation profile had not improved. Although the platelet count was 113 × 10^9^ cells/L on admission, the count began decreasing since Day 25 till it reached 73 × 10^9^ cells/Lon Day 31. Therefore, to further explore the cause of the coagulation abnormality and continue the treatment of CMV colitis, the patient was transferred to our hospital on Day 32.

**Fig. 3:**
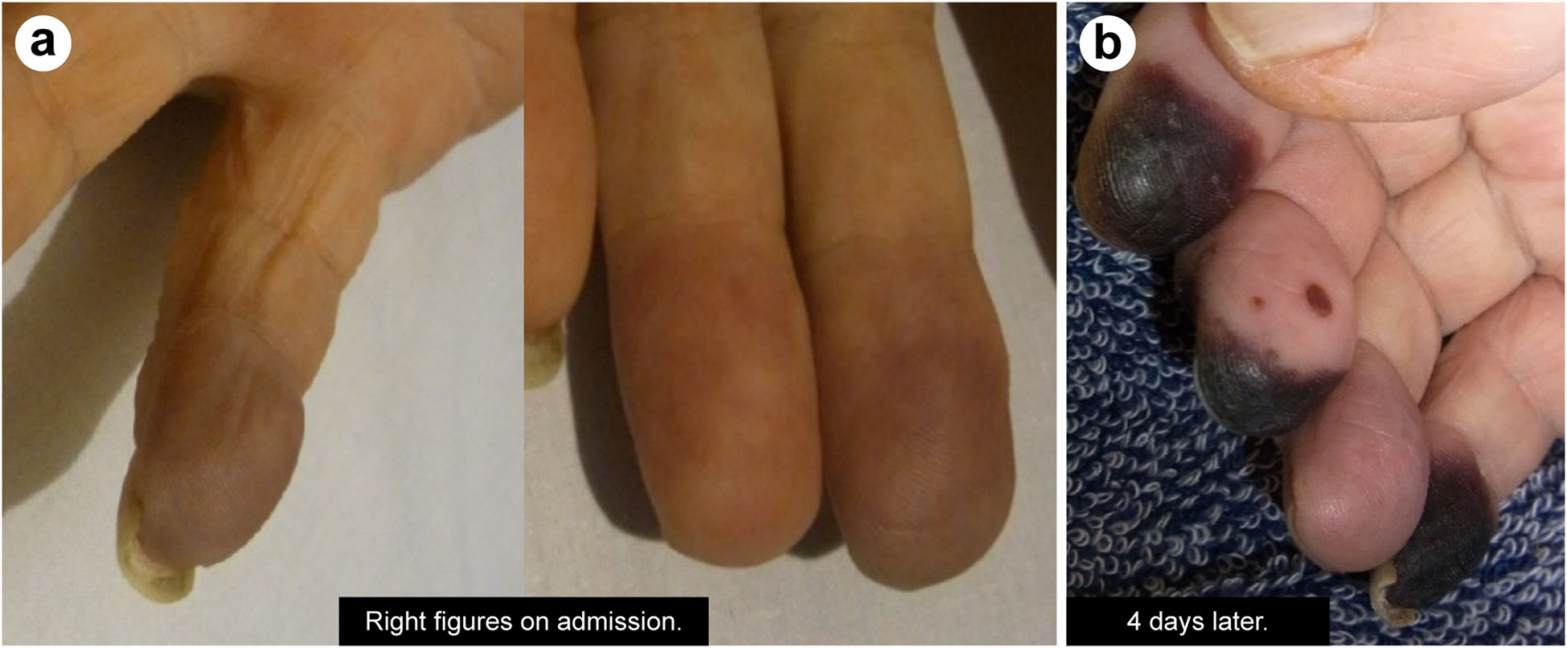
Purpura on the apex of the fingers at the time of admission. In the right hand, there is an enlargement of the purpura

On admission to our facility, the patient’s clinical findings were as follows: Height, 163 cm; weight, 51 kg; body temperature, 36.8 °C; pulse rate, 108 beats/min; blood pressure, 118/92 mmHg; respiratory rate, 12 breaths/min; and oxygen saturation, 90% on ambient air. Breathing sounds were heard with coarse cracks on the dorsal surface of the lungs bilaterally. The heartbeat was irregular, but no heart murmur was heard. The abdomen was flat and soft, and there was no spontaneous pain; however, there was tenderness on palpation in the right hypochondrium and iliac regions. No peritoneal irritation was observed. Bilateral to the chest, approximately 20 scattered erythematous papules (~ 1 cm in length) were found; no superficial lymph nodes were palpable. Purpuric spots and pain were present on the right fifth finger, whereas only purpuric spots were seen on the left second and third fingers (Fig. 3). A peripherally inserted central catheter that was placed in the left upper arm at the previous facility was present.

Table 1 shows the patient’s laboratory data upon his admission to our hospital (Day 35 from the day of admission at the first facility).

**Table 1:** Laboratory data of the patient upon admission to our hospital

Parameter	Recorded value	Standard value
White blood cell count, cells/L	14.54 × 10^9^	4.5–7.5 × 10^9^
Neutrophils, %	34.0	42–74
Eosinophil, %	50.5	0–7.0
Hemoglobin, g/L	102	113–152
Platelet count, cells/L	6 × 10^9^	130–350 × 10^9^
Prothrombin time/International normalized ratio	4.37	0.80–1.20
Activated partial thromboplastin time, s	154.1	26.9–38.1
D-dimer, mg/L	2.1	< 1.0
Fibrin degradation product, mg/L	3.6	0–5.0
Fibrinogen, g/L	2.97	2.00–4.00
Antithrombin III, %	37.2	70–120
C-reactive protein, nmol/L	184.77	≤5.71
Total protein, g/L	71	69–84
Albumin, g/L	18	39–51
Total bilirubin, mmol/L	0.0308	0.0034–0.0205
Aspartate aminotransferase, μkat/L	0.32	0.18–0.5
Alanine aminotransferase, μkat/L	0.47	0.07–0.5
Lactase dehydrogenase, μkat/L	4.55	1.8–3.6
alkaline phosphatase, μkat/L	25.02	1.67–5.83
γ- glutamyltransferase, μkat/L	1.62	0.17–0.83
Creatine kinase, μkat/L	0.93	0.67–2.5
Blood urea nitrogen, mmol/L	9.43	2.86–7.14
Creatinine, mmol/L	0.06	0.05–0.09
Sodium, mmol/L	136	136–148
Potassium, mmol/L	4.5	3.6–5.0
Chloride, mmol/L	107	98–108
soluble interleukin-2 receptor, U/mL	8540	140–520
IgG4, μmol/L	28.62	0.33–7.80
IgE, U/mL	20,367	≤173
P-ANCA	–	
C-ANCA	–	
Antinuclear antibody	40 times	< 80 times
C7-HRP	0	
TAT, ng/mL	2.6	0.0–3.9
PIC, μg/mL	< 0.2	0.0–0.8
Haptoglobin, mg/dL	126	71–160
Activation of factor II, %	49.1	66–118
Activation of factor V, %	< 1.0	73–122
Activation of factor X, %	59.7	58–200
Protein C, %	37%	70–140%
Protein S, %	64.1%	63–149%
Cardiolipin antibody IgG, U/mL	25	0–9
Cardiolipin antibody IgM, U/mL	3	0–7
Anti-human beta-2 glycoprotein 1 Antibody, U/mL	< 1.3	0.0–3.4
lupus anticoagulant	Unknown	0.0–1.2
Activation of ADAMTS13, %	18	≥78

*C-ANCA* cytoplasmic anti-neutorophil antibody, *C7-HRP* cytomegalovirus pp65 antigen, *P-ANCA* perinuclear anti-neutorophil antibody, *PIC* plasmin-α2 plasmin inhibitor complex, *TAT* thrombin anti-thrombin complex

These were as follows: White blood cell count, 14.54 × 10^9^ cells/L; C-reactive protein, 1847.62 nmol/L; platelet count, 60 × 10^9^ cells/L; and normocytic anemia. There was no significant abnormality in the peripheral blood; however, biliary enzymes were elevated (alkaline phosphatase: 20.02 μkat/L; γ- glutamyltransferase: 1.62 μkat/L). The coagulation disorder was apparent (PT-INR, 4.37; APTT, 154.1 s) but the D-dimer level was virtually unchanged (2.1 mg/L) and the level of fibrin degradation products was normal (3.6 mg/mL). Levels of thrombin-antithrombin complex (2.6 ng/mL) and plasmin-α2 plasmin inhibitor complex (< 0.2 ng/mL) were also normal. However, soluble interleukin-2 receptor (sIL-2R) was elevated (8540 U/mL), factor II activity was marginally reduced (49.1%), factor X activity was reduced to 59.7%, factor V activity was < 1.0%, and ADAMTS13 activity had decreased to 18%. Abdominal CT showed splenomegaly with a maximum splenic length of 9.6 cm and enlargement of axillary, left superior fossa, and paraortic lymph nodes. On Day 1, the patient had a maximum splenic length of 7.6 cm, indicating that the splenomegaly had increased since then.

**Fig. 4:**
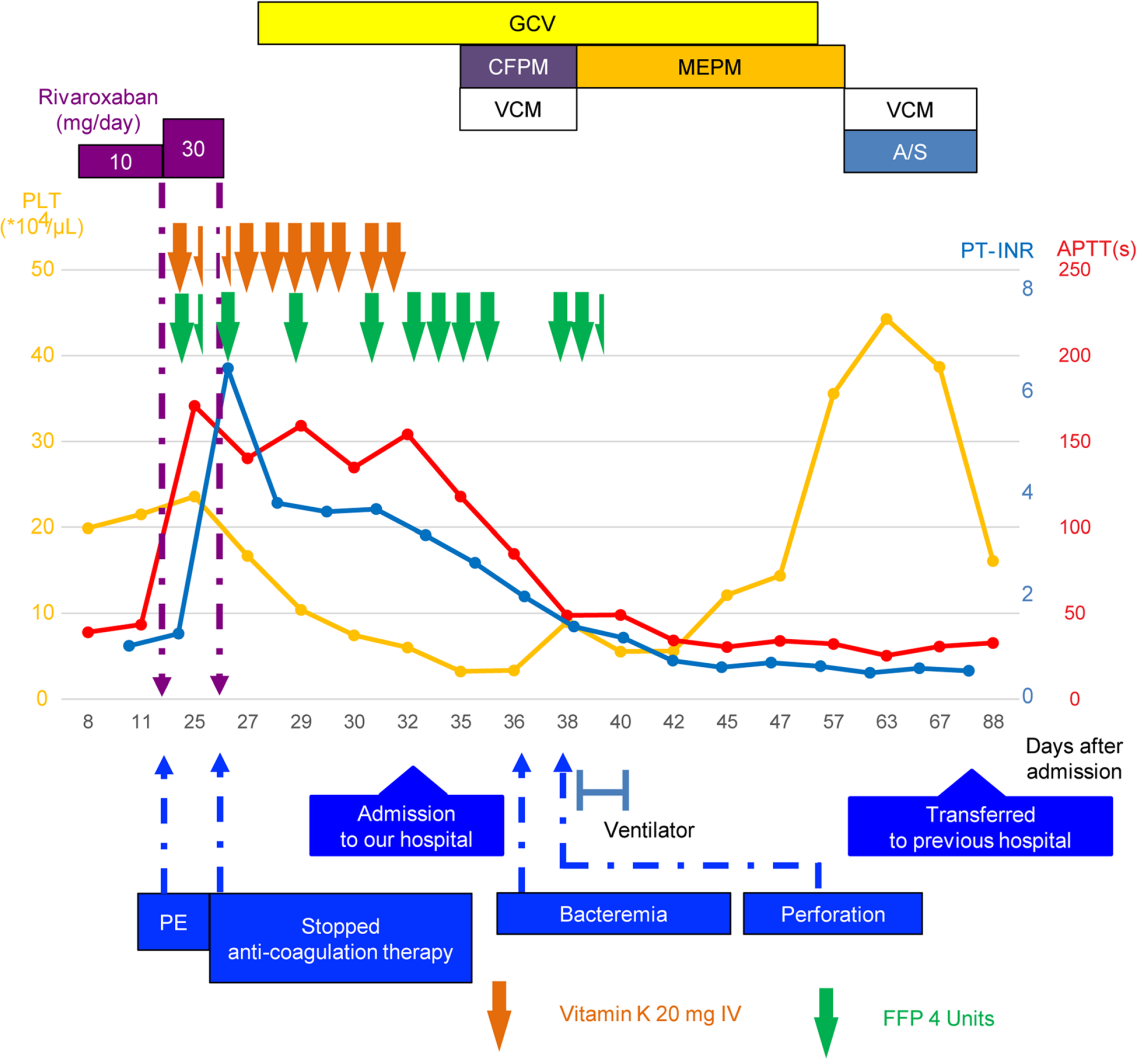
Chart showing the clinical course after admission. PE: pulmonary embolism; GCV: ganciclovir; CFPM: cefepime; MEPM: meropenem; VCM: vancomycin; A/S: Ampicillin/Sulbactam

Figure 4 shows the clinical course after admission at the first facility. The administration of 5 mg/kg GCV every 12 h was continued. The purpura on the right hand observed at the time of admission to our facility widened. A total of 16 units of FFP were transfused to the patient at our hospital for 3 days (from Day 35, Day 4 from the admission to our hospital) but the coagulation profile did not improve. Since this patient had CMV enteritis, we considered vasculitis or malignant lymphoma as the cause based on the purpura on the fingertips, truncal rash, increasing splenomegaly, high alkaline phosphatase (ALP) level, high sIL-2R level, low platelet count, and PT-INR and APTT prolongation. A biopsy of the skin rash on the right lateral surface of the abdomen and of a left axillary lymph node was performed on Day 34 and Day 36 (Day 3 and 5 from the admission to our hospital), respectively. However, there were no indications of vasculitis or malignant lymphoma.

**Fig. 5:**
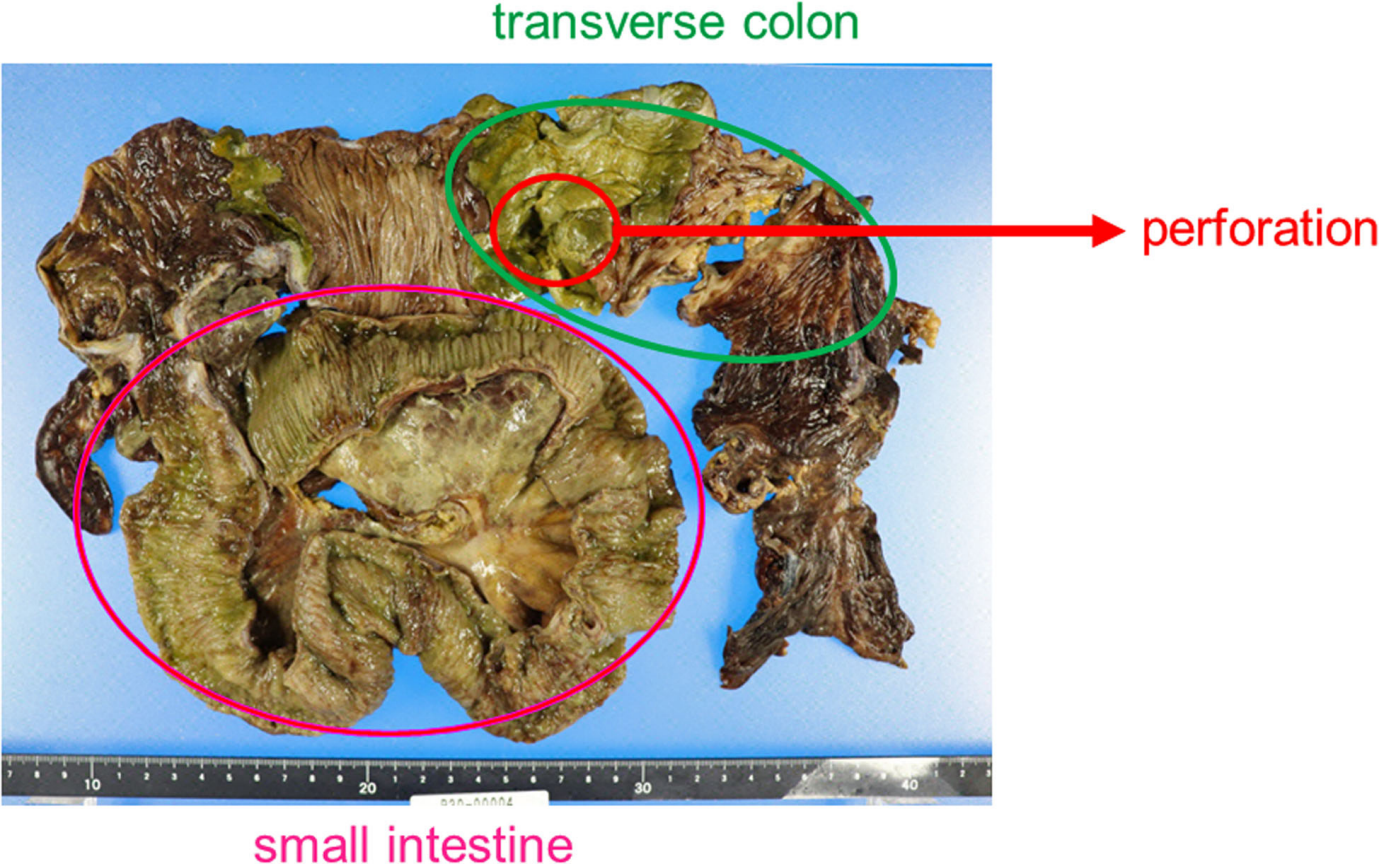
Resected intestinal tract. A perforation was found 10 cm to the right from the top of the transverse colon

**Fig. 6:**
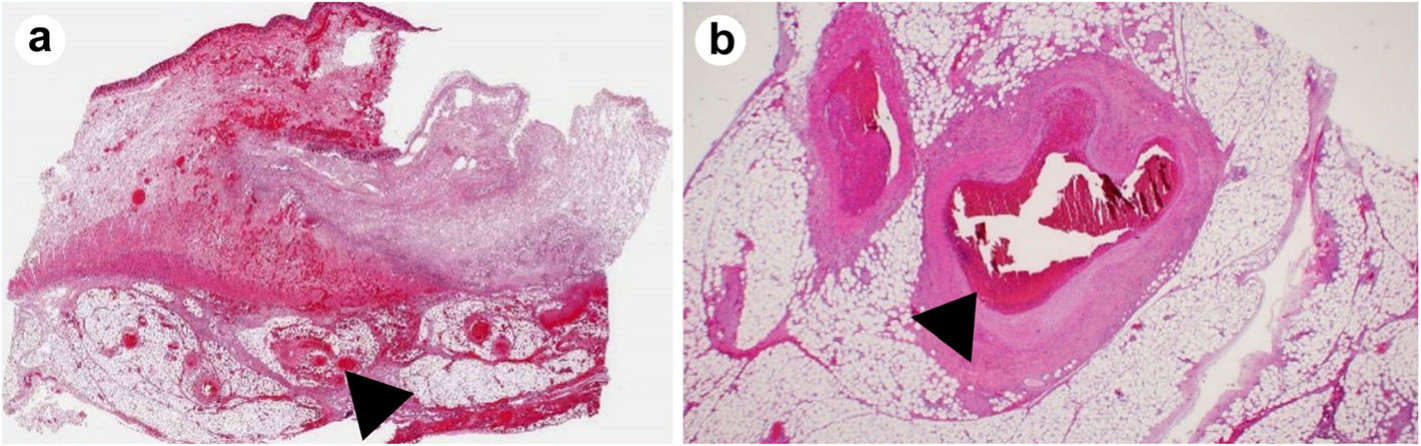
Hematoxylin-eosin stained resected intestinal tract (× 2). An abundance of thrombi in small and medium-sized arteriovenous ducts (arrows) was found

On Day 35 (Day 4 from the admission to our hospital), the patient developed bacteremia caused by *Staphylococcus aureus* and *Fusobacterium* species. He was treated with meropenem (MEPM) 2 g/day and vancomycin (VCM; trough ≥15 μg/mL). On Day 37 (Day 6 from the admission to our hospital), his abdominal pain worsened, and he went into shock. Non-contrast CT of the abdomen showed extensive free air in the abdominal cavity, which led to the diagnosis of gastrointestinal perforation that warranted an emergency surgery. During the operation, scattered necrotic changes were observed in the small intestine. A 1-cm perforation was found 10 cm to the right from the top of the transverse colon; the colon was resected from the ileum to the perforated area (Fig. 5). An additional excision was performed on the left side of the transverse colon because of stool drainage from that region. Subsequently, ileal colostomy was performed, and a stoma was placed in the right lower abdomen. The surgical specimen of the ileum showed a perforation in the transverse colon, necrosis due to ischemic changes associated with circulatory failure, and numerous thrombi, including microthrombi, in the small- and medium-sized arteriovenous ducts (Fig. 6). There was no evidence of lymphoma, vasculitis, or ulcerative colitis on pathological examination. We performed cardiac ultrasound scan multiple times, but no valve destruction was seen. In addition, a second blood culture was negative. Abnormal coagulation and multiple thrombosis were present before the onset of staphylococcal bloodstream infection. Furthermore, the pathology of the intestine showed no invasion by *S. aureus*. Therefore, we thought that Staphylococcal bloodstream infection had little to do with abnormal coagulation and multiple thrombosis. Because of the drug sensitivities of *S. aureus* and *Fusobacterium* species and the presence of intestinal perforation, VCM was discontinued, and only MEPM (1 g, 12 hourly) was administered from Day 39 (Day 8 from the admission to our hospital) onward. The thrombocytopenia and coagulation abnormalities observed preoperatively improved slightly and normalized postoperatively. In addition, the activities of factor II, factor X, factor V, and ADAMTS13 normalized. There was no evidence of vasculitis, lymphoma, or any other thrombotic disease; therefore, the final diagnosis was multiple microthrombi caused by CMV enteritis that mimicked thrombotic microangiopathy-like conditions. GCV administration was continued until Day 57 (Day 26 from the admission to our hospital).

On Day 88 (Day 57 from the admission to our hospital), the patient was transferred to the previous hospital for rehabilitation. One year has passed since he was discharged from the hospital; no recurrence has been observed since.

## Discussion and conclusions

We presented a case of multiple microthrombosis triggered by CMV enterocolitis in a patient without an underlying disease that causes immunodeficiency. This is an extremely rare case that ADAMTS13 activity was reduced, resulting in multiple microthrombi and a thrombotic microangiopathy-like condition in the patient with CMV infection.

**Fig. 7:**
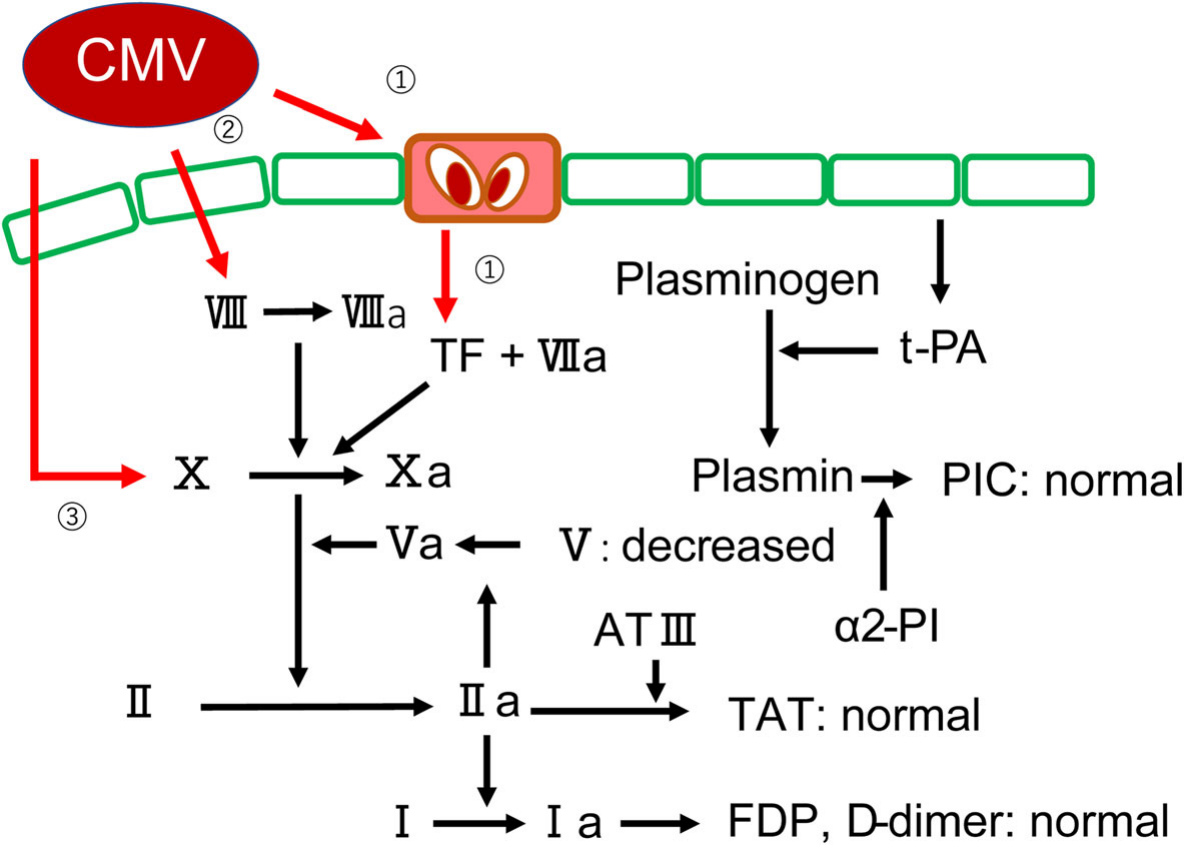
Coagulation cascade and interference of cytomegalovirus in this case. 1) Stimulation of endovascular cells. As a result, production of TF and vVF are decreased and activation of ADAMTS13 is decreased. 2) Production of factor VII. 3) Activation of factor X

Arteriovenous thrombosis has been reported as a rare complication of CMV infection [4, 5]. As shown in Fig. 7, the mechanism that contributes to the formation of a thrombus due to CMV infection has been reported [1] to stimulate vascular endothelial cells, promote the production of tissue factor and von Willebrand factor (vWF) [2], produce factor VIII, and [3] activate factor X [1]. To date, approximately 100 cases have been reported [1], most of which report the presence of relatively large single thrombi in sites such as deep veins, pulmonary arteries, or visceral arteriovenous ducts [5, 6]. Multiple thrombi are extremely rare in such cases. To the best of our knowledge, there are only two similar cases that report multiple microthrombi as a consequence of CMV infection [2, 7]. One case is that of thrombotic thrombocytopenic purpura (TTP) in an immunocompromised individual [2] and the other is of hemolytic-uremic syndrome (HUS) in an immunocompetent individual [7]. In the presented case, however, no fragmented red blood cells were observed in the peripheral blood, but laboratory data showed a marked decrease in the platelet count and ADAMTS13 activity (baseline value, > 78%). The diagnosis of a TMA-like pathology was made based on the necrosis of the fingers and the presence of microthrombi in the intestinal tract in the surgical specimen. However, differing from previously reported pathogenesis of TTP, the ADAMTS13 activity was not < 10% [8]. Moreover, the condition differed from HUS because Shiga toxins were not involved and kidney failure did not occur [9, 10]. We believe that ADAMTS13 was markedly reduced in the present case as a result of the presumed mechanism of the stimulation of vascular endothelial cells and promotion of excessive tissue factor and vWF production [1]. The treatment of simple large thrombosis, such as pulmonary embolism and superior mesenteric venous embolism, associated with CMV infection is anticoagulation therapy with heparin or warfarin [5] in conjunction with antiviral medication such as GCV to treat the CMV infection [11].

The hyper prolongation of PT-INR and APTT in the presented case was initially suspected to be due to an exhaustive coagulopathy caused by an increased secondary hemostatic mechanism like coagulation factor activation or a disseminated intravascular coagulation-like condition. However, there was no hyper prolongation of other coagulation systems, such as thrombin-antithrombin complex, plasmin-α2 plasmin inhibitor complex, and fibrin degradation products, and D-dimer was not elevated (Fig. 7). The activity of factor V, a cofactor of the secondary hemostatic cascade, was greatly reduced, indicating that the cause was an acquired factor V deficiency due to the action of factor V inhibitors. Factor V deficiency is the second most common form of acquired hemophilia [12], with a reported incidence of 0.09 to 0.29 per million people per year [13], and has several causes, including viral infections, collagen diseases, malignancies, medicines, diabetes mellitus, and inflammatory bowel disease [14]. We theorize that an acquired factor V deficiency was triggered by the CMV infection in the present case because PT-INR, APTT, and coagulation factors normalized after the CMV infection was cured.

Most adults are subclinically infected with CMV during childhood and remain latently infected throughout their lives without developing noticeable symptoms or pathology in normal immune conditions. However, immunosuppressive conditions, such as acquired immunodeficiency syndrome, post-organ transplantation, and reception of immunosuppressive medicines such as anticancer drugs/corticosteroids, may lead to CMV reactivation that may cause opportunistic infections [15, 16]. Further, the management of patients affected by autoimmune/idiopathic diseases has been revolutionized by the development of targeted therapies in recent years. However, targeted therapies are also a risk for CMV reactivation [17]. In the present case, although the patient was regularly inhaling formoterol fumarate for managing bronchial asthma, there was no preexisting disease that could cause immunosuppression. Thrombosis associated with CMV infection often occurs in immunocompromised individuals [1, 18], but in recent years, it has been reported in immunocompetent patients [4, 19] as well. In the present case, as colon cancer was initially suspected, lower gastrointestinal endoscopy was delayed until Day 14 and was not performed immediately after hospitalization. In addition, it took 2 weeks to confirm the pathological diagnosis. Therefore, GCV treatment was delayed as it commenced on Day 27 after admission.

The eosinophil percentage was 50% at admission, gradually improved, and normalized with improvement of the coagulation abnormalities after surgery. In addition to bronchial asthma as one of the underlying diseases, coagulation abnormalities associated with infection are thought to be involved in eosinopenia, but the actual cause is unknown.

In conclusion, we present an extremely rare case wherein the ADAMTS13 activity was reduced, resulting in a thrombotic microangiopathy-like condition in an immunocompetent patient with CMV enterocolitis. Our findings suggest that CMV infection may be considered as a differential diagnosis when treating immunocompetent individuals who present with thrombosis of an unspecified cause.

## Data Availability

Data sharing is not applicable to this article as no datasets were generated or analyzed during the current study.

## Abbreviations

ALP: Alkaline phosphatase
APTT: Activated partial thromboplastin time
CMV: Cytomegalovirus
CT: Computed tomography
FFP: Fresh-frozen Plasma
GCV: Ganciclovir
HUS: Hemolytic-uremic syndrome
MEPM: Meropenem
PT-INR: International normalized ratio of prothrombin time
sIL-2R: Soluble interleukin-2 receptor
TMA: Thrombotic microangiopathy
TTP: Thrombotic thrombocytopenic purpura
VCM: Vancomycin
vWF: von Willebrand factor
